# Preoperative chemoradiotherapy in older patients with rectal cancer guided by comprehensive geriatric assessment within a multidisciplinary team—a multicenter phase II trial

**DOI:** 10.1186/s12877-024-05046-6

**Published:** 2024-05-21

**Authors:** Wen-Yang Liu, Yuan Tang, Ning Li, Yu Tang, Yun-Jie Cheng, Lin Yang, Hui Fang, Ning-Ning Lu, Shu-Nan Qi, Bo Chen, Shu-Lian Wang, Yong-Wen Song, Yue-Ping Liu, Ye-Xiong Li, Zheng Liu, Jian-Wei Liang, Wei Pei, Xi-Shan Wang, Hai-Zeng Zhang, Jun Wang, Hai-Tao Zhou, Jing Jin

**Affiliations:** 1 Department of Radiation Oncology, National Cancer Center/National Clinical Research Center for Cancer/Cancer Hospital, Chinese Academy of Medical Sciences and Peking Union Medical College, Beijing, China; 2 Department of Radiation Oncology, National Cancer Center/ Cancer Hospital & Shenzhen Hospital, Chinese Academy of Medical Sciences and Peking Union Medical College, Shenzhen, China; 3 Department of Colorectal Surgery, National Cancer Center/National Clinical Research Center for Cancer/Cancer Hospital, Chinese Academy of Medical Sciences and Peking Union Medical College, Beijing, China; 4 Department of Medical Oncology, National Cancer Center/National Clinical Research Center for Cancer/Cancer Hospital, Chinese Academy of Medical Sciences and Peking Union Medical College, Beijing, China; 5 Clinical Trials Center, National Cancer Center/National Clinical Research Center for Cancer/Cancer Hospital, Chinese Academy of Medical Sciences and Peking Union Medical College, Beijing, China; 6 Department of Radiation Oncology, The Fourth Hospital of Hebei Medical University, Shijiazhuang, China

**Keywords:** Rectal cancer, Preoperative chemoradiotherapy, Comprehensive geriatric assessment, Geriatric oncology

## Abstract

**Background:**

The purpose of this study was to evaluate the safety and efficacy of preoperative concurrent chemoradiotherapy (preCRT) for locally advanced rectal cancer in older people who were classified as “fit” by comprehensive geriatric assessment (CGA).

**Methods:**

A single-arm, multicenter, phase II trial was designed. Patients were eligible for this study if they were aged 70 years or above and met the standards of “fit” (SIOG1) as evaluated by CGA and of the locally advanced risk category. The primary endpoint was 2-year disease-free survival (DFS). Patients were scheduled to receive preCRT (50 Gy) with raltitrexed (3 mg/m2 on days 1 and 22).

**Results:**

One hundred and nine patients were evaluated by CGA, of whom eighty-six, eleven and twelve were classified into the fit, intermediate and frail category. Sixty-eight fit patients with a median age of 74 years were enrolled. Sixty-four patients (94.1%) finished radiotherapy without dose reduction. Fifty-four (79.3%) patients finished the prescribed raltitrexed therapy as planned. Serious toxicity (grade 3 or above) was observed in twenty-four patients (35.3%), and fourteen patients (20.6%) experienced non-hematological side effects. Within a median follow-up time of 36.0 months (range: 5.9-63.1 months), the 2-year overall survival (OS), cancer-specific survival (CSS) and disease-free survival (DFS) rates were 89.6% (95% CI: 82.3-96.9), 92.4% (95% CI: 85.9-98.9) and 75.6% (95% CI: 65.2-86.0), respectively. Forty-eight patients (70.6%) underwent surgery (R0 resection 95.8%, R1 resection 4.2%), the corresponding R0 resection rate among the patients with positive mesorectal fascia status was 76.6% (36/47).

**Conclusion:**

This phase II trial suggests that preCRT is efficient with tolerable toxicities in older rectal cancer patients who were evaluated as fit based on CGA.

**Trial registration:**

The registration number on ClinicalTrials.gov was NCT02992886 (14/12/2016).

**Supplementary Information:**

The online version contains supplementary material available at 10.1186/s12877-024-05046-6.

## Background

The patient population with rectal cancer is predominantly of older age [1, 2]. However, the choice of treatment regimen is a challenging decision for these patients because older patients are rarely enrolled in specific prospective trials [3].

For older patients with locally advanced rectal cancer, preoperative concurrent chemoradiotherapy (preCRT) is the preferred option if they are considered fit according to several consensuses [3, 4]. Despite the recommendations from these publications, the evidence mostly comes from retrospective studies or subgroup analyses of prospective trials [5–7], until the recent release of one randomized controlled trial (RCT) PRODIGE 42/GERICO 12 study comparing short course radiotherapy with chemoradiotherapy for locally advanced rectal cancer (LARC) in the older patients [8]. Still, geriatric assessment outcome was analyzed for change after treatment, but not as specific stratification tool for decision making in the PRODIGE 42/GERICO 12 study. Consequently, to date, there are no robust data to support the appropriate therapy for LARC patients with a particular status as judged by a geriatric assessment tool [9].

A multidisciplinary team (MDT) is essential for developing the complex treatment required by rectal cancer[10]. The frailty assessment is also important [11]. Comprehensive geriatric assessment (CGA) is the most widely used evaluation method and is strongly recommended by the International Society of Geriatric Oncology for older cancer patients [12]. Although time consuming, this tool provides an exhaustive assessment of major dimensions of frailty, including but not limited to comorbid conditions, nutritional status, and cognitive performance. A previous study indicated that CGA could predict the safety of surgery in patients with colorectal cancer [13]. Recently, multiple randomized trials have demonstrated that CGA can help tailor regimens [14] and decrease cancer treatment toxicity [15, 16]. Nevertheless, in rectal cancer, CGA is seldom investigated for its impact on decision-making [3]. One randomized trial included frail older patients to receive preoperative GA and accordingly tailored interventions only for surgery; unfortunately, Grade II–V complications were not reduced in these patients who underwent elective surgery for colorectal cancer (CRC) [17].

Without a CGA evaluation, our previous phase I trial in rectal cancer patients aged 75 years indicated higher levels of toxicity from preCRT, and surprisingly, the planned surgery was conducted in less than half of the patients [18]. In addition, poor mucositis tolerance and more cardiovascular comorbidities were observed in these patients. Consequently, this multicenter phase II trial, guided by MDT and CGA was designed to validate the concept that “fit” older patients with LARC can receive the same standard of care as younger counterparts. In this study, Raltitrexed was combined with concurrent radiotherapy because of its lower incidence of inducing mucositis and cardiac toxicity [19, 20], along with non-inferior efficacy compared to 5-fluorouracil/leucovorin [21]. Our prior finding in the interim analysis showed that preCRT is well tolerated with high compliance in fit older patients [22]. Here, we report the primary endpoint of 2-year disease-free survival (DFS) according to the TREND statements (https://www.cdc.gov/trendstatement/pdf/trendstatement_trend_checklist.pdf).

## Methods

### Trial design and participants

Details of the design and implementation of this study have been previously reported [22]. Briefly, eligible patients met the criteria of being fit according to the standard of SIOG1 [23], with no evidence of serious comorbidity (CISR-G Grade 0, 1 or 2), no dependence in IADL and ADL or malnutrition, and those aged 70 years or above. Meanwhile, their rectal adenocarcinoma fulfilled the standard of locally advanced risk category (bad and ugly) defined by the European Society for Medical Oncology (ESMO) Clinical Practice Guidelines [10]. All patients were evaluated carefully and identified as candidates for preCRT by the MDT.

### Procedures

CGA was conducted for all participants by a geriatric oncologist with more than 5 years of experience in CGA after written informed consent was provided. And the components of the CGA were presented in Table 2. Among them, the social support score (low, ≤44; and high, >44) was calculated using the tool developed by Xiao et al and classified according to its guidelines [24]. This system consists of 10 items across three dimensions: objective support (low, ≤13; and high, >13), subjective support (low, ≤24; and high, >24), and support utilization (low, ≤13; and high, >13). Four-point scoring (refuse=1; somewhat not willing=2; somewhat willing=3; very willing=4) was used to evaluate the willingness of patients and their families to undergo surgery. Chest and abdominal computed tomography, endoscopic ultrasound (EUS), and/or pelvic magnetic resonance imaging (MRI) were used for clinical staging evaluation (according to the AJCC 7th edition). Except for patients who refused preCRT, all patients who met the inclusion criteria were enrolled in the study.

Preoperative chemoradiotherapy with raltitrexed was delivered to the patients, followed by surgery. Radiotherapy was delivered to a planning target volume (in accordance with the International Consensus [25]) with a dose of 50 Gy (2.0 Gy daily, 5 days per week) with intensity-modulated radiotherapy or volumetric-modulated arc therapy. Image guided radiotherapy was done with cone beam computed tomography daily in the first five fractions and subsequently once a week. Chemotherapy was administered concurrently (Raltitrexed, intravenous infusion, 3 mg/m^2^ on days 1 and 22).

### Endpoints

The primary endpoint was DFS, which was defined as theinterval between inclusion and the recurrence or death from any cause. The secondary end points included overall survival (OS, time from the end of preCRT or surgery to death because of any cause), cancer-specific survival (CSS, time from the end of preCRT or surgery to death because of cancer), the ratio of patients occured pathologic complete response (pCR) and the ratio of patients occurred Grade 3 or higher adverse events (During chemoradiotherapy and within 180 days after surgery). The National Cancer Institute Common Terminology Criteria for Adverse Events version 4.0. was used for toxicity assessment, and the Clavien–Dindo system was employed for evaluating surgery-related complications. Exploratory endpoints included investigating the CGA elements, and willingness evaluation to surgery for predicting the adherence.

### Statistical analysis

Fifty-one patients were required to test the hypothesis that the 2-year DFS was equal to or greater than 78% (This value was based on the data from five large European rectal cancer trials) [26], if the lower bound of 95% CI for 2-year DFS in this study is greater than 63%, with 80% power at a significance level of 5% (one-sided) to reject the null hypothesis, then the study treatment would be considered efficient. Considering a 5% drop-out rate and a 20% rate of surgery refusal, 68 patients had to be included.

Analysis was based on the intention-to-treat principle. Given the instability of older patients' adherence to surgery, per-protocol population (PP) was prospectively defined as those who were willing and attempted to undergo surgery (whether successful or not) or were evaluated as having a complete clinical response (CCR) and deemed suitable for the watch-and-wait strategy, in order to gather more information.

Survivals were analyzed with the Kaplan-Meier method. Evaluation of the factors influencing the compliance of patients for surgery was analyzed by multivariate logistic regression. Frequency was used to describe the toxicities and treatment completion rate. All statistical analysis except the primary endpoint hypothesis were tested at a two-sided significance level of 0.05. Calculations were conducted by IBM SPSS Statistics for Windows, Version 23.0. Armonk, NY: IBM Corp.

## Results

### Participants

**Fig. 1:**
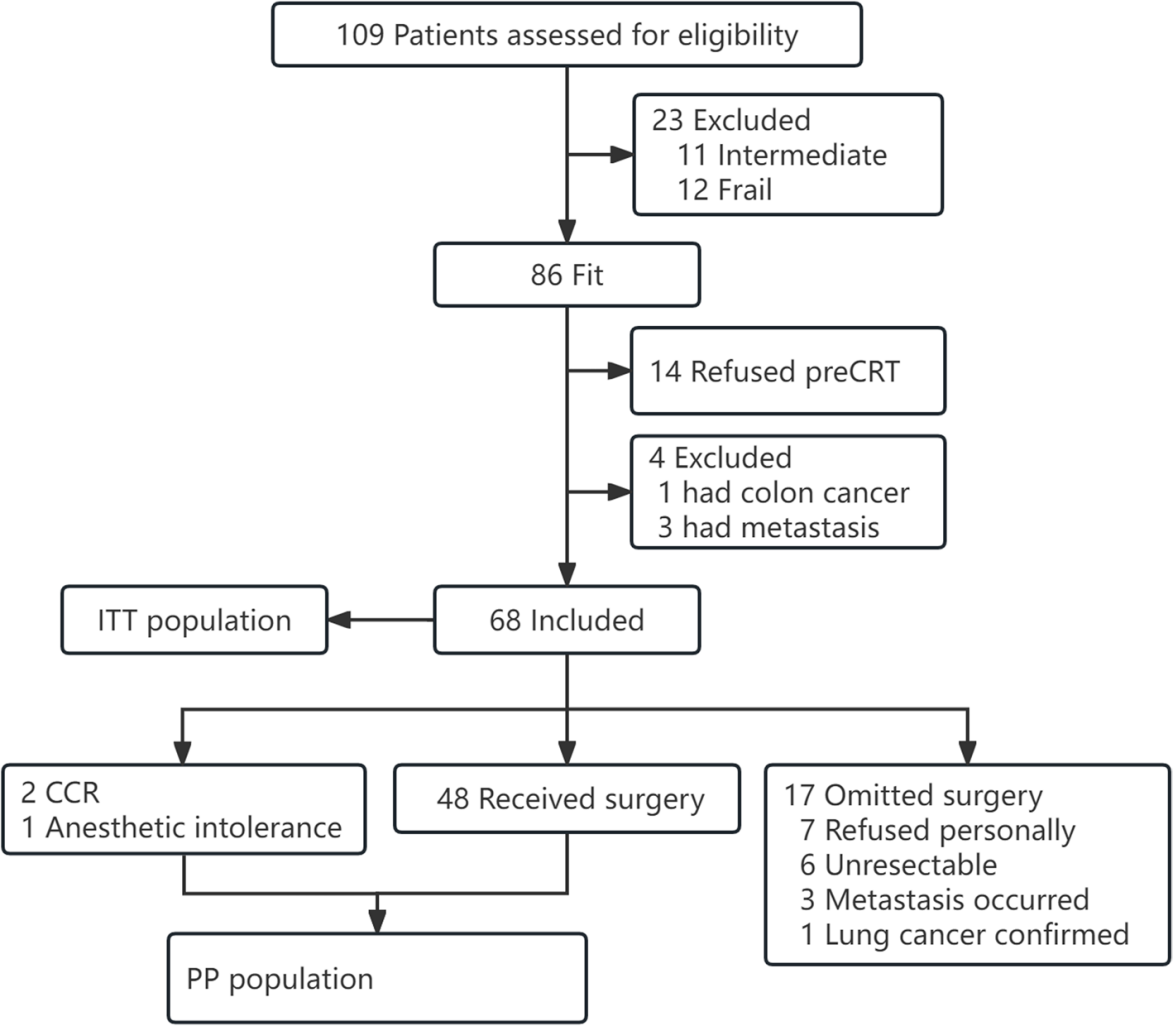
CONSORT diagram. Abbreviations: preCRT, preoperative chemoradiotherapy, ITT, intention-to-treat, CCR, complete clinical response, PP, per-protocol

Between Sep. 2016 and Oct. 2019, from two cancer centers in China, 109 patients were evaluated by the MDT and CGA, of whom 86, 11, and 12 were classified into the fit, intermediate and frail category, respectively. Among these fit patients, 68 were enrolled into this trial (Fig. 1). The intention-to-treat (ITT) population (68) was analyzed for the primary endpoint and safety. The per-protocol (PP) population (51) was also evaluated for the primary endpoint as planned. Patients had a median age of 74 years (range 72-77). Hypertension or cardiovascular disease (48.5%), and diabetes (19.1%) were the most common comorbidities. The tumor characteristics and CGA evaluation of the patients are shown in Table 1 and Table 2, respectively. In 55 (80.1%) patients, at least one high-risk factor was observed on pelvic MRI (with at least one of the following criteria: clinical tumour [cT] stage cT4a or cT4b, extramural vascular invasion, clinical nodal [cN] stage cN2, involved mesorectal fascia, or enlarged lateral lymph nodes).

**Table 1:** Baseline characteristics of patients included in this study

Parameters	ITT population No. (%) (*N*=68)	PP population No. (%) (*N*=51)
Age in years, median (range)	74 (72-77)	73 (71-76)
71-74	37 (54.4)	29 (56.9)
75-79	25 (36.8)	19 (37.3)
80-89	6 (8.8)	3 (5.9)
Gender		
Female	25 (36.8)	16 (31.4)
Male	43 (63.2)	35 (68.6)
Tumor stage evaluation		
Pelvic MRI	65 (95.6)	49 (96.1)
EUS and pelvic CT	3 (4.4)	2 (3.9)
Primary tumor Stage		
T2	2 (2.9)	0
T3	49 (72.1)	40 (78.4)
T4a	10 (14.7)	7 (13.7)
T4b	7 (10.3)	4 (7.8)
Nodal stage		
N0	16 (23.5)	16 (31.4)
N1a-b	34 (50.0)	16 (31.4)
N2a-b	28 (26.5)	19 (37.3)
MRF status		
Negative	20 (29.4)	26 (51.0)
Positive	47 (69.1)	24 (47.1)
NA	1 (1.5)	1 (2.0)
EMVI		
Negative	46 (67.6)	31 (60.8)
Positive	19 (27.9)	18 (35.3)
NA	3 (4.4)	2 (3.9)
Distance from anal verge (cm)		
≤5	49 (72.1)	34 (66.7)
5-10	17 (25.0)	15 (29.4)
11	2 (2.9)	2 (3.9)
Histology		
Adenocarcinoma (NOS)	22 (32.4)	19 (37.3)
Well differentiated adenocarcinoma	3 (4.4)	1 (2.0)
Moderately differentiated adenocarcinoma	35 (51.5)	28 (54.9)
Poorly differentiated adenocarcinoma	7 (10.3)	3 (5.9)
Mucinous adenocarcinoma	1 (1.5)	0

*Abbreviations*: *ITT* intention-to-treat, *PP* per-protocol, *MRI* magnetic resonance imaging, *EUS* endoscopic ultrasound, *CT* computed tomography, *MRF* mesorectal fascia, *EMVI* Extramural vascular invasion, *NOS* not otherwise specified

**Table 2:** Baseline geriatric assessment of patients

Geriatric Parameters	ITT population No. (%) (*N*=68)	PP population No. (%) (*N*=51)
KPS		
90	1 (1.5)	1 (2.0)
80	63 (92.6)	49 (96.0)
70	4 (5.9)	1 (2.0)
CCI		
0	46 (67.6)	35 (68.6)
1	17 (25.0)	12 (23.5)
2	5 (7.4)	4 (7.9)
CIRS-G score		
0	15 (22.1)	10 (19.6)
1-3	33 (48.5)	25 (49.0)
4-6	20 (29.4)	16 (31.4)
ADL		
100	55 (80.9)	41 (80.4)
95	7 (10.3)	6 (11.8)
90	6 (8.8)	4 (7.8)
IADL		
8	65 (95.6)	49 (96.1)
7	2 (2.9)	2 (3.9)
6	1 (1.5)	0
MNA		
≥24	49 (72.1)	41 (80.4)
17-24	19 (27.9)	10 (19.6)
TUG		
≤10	58 (85.3)	44 (86.3)
11-15	10 (14.7)	7 (13.7)
GDS15		
≤4	65 (95.6)	48 (94.1)
4-8	2 (2.9)	2 (3.9)
>8	1 (1.5)	1 (2.0)
MMSE		
26-30	57 (83.8)	44 (86.3)
20-25	11 (16.2)	7 (13.7)
Social support		
Low	58 (85.3)	43 (84.3)
High	10 (14.7)	8 (15.7)
Objective support		
Low	61 (89.7)	45 (88.2)
High	7 (10.3)	6 (11.8)
Subjective support		
Low	60 (88.2)	43 (84.3)
High	8 (11.7)	8 (15.7)
Support utilization		
Low	39 (57.4)	31 (60.8)
High	29 (42.6)	20 (39.2)

*Abbreviations:* *ADL* Activities of daily living, *CCI* Charlson Comorbidity Index, *CGA* Comprehensive geriatric assessment, *CIRS-G* Cumulative Illness Rating Scale for Geriatrics, *GDS15* Geriatric Depression Scale 15, *IADL* Instrumental activities of daily living, *MMSE* Mini-Mental State Examination, *MNA* Mini Nutritional Assessment, *PS* performance status, *TUG* Timed "Up & Go"

### Treatment compliance

During the preoperative phase, 94.1% and 79.3% of the patients finished the prescription RT dose and concurrent chemotherapy as scheduled, respectively (five patients received 1650 mg/m^2^ capecitabine, three due to physician error and the other two due to the convenience of outpatient oral administration, Table 3). Among the 58 candidates for surgery after preCRT, seven personally refused the operation. Of the remaining 10 patients not considered for surgery, there were six due to unresectable disease, three due to distant metastasis and one due to lung cancer confirmed.

**Table 3:** Preoperative CRT and surgery completion profile

Items	No. (%) (*N*=68)
RT	
Dose delivered as planned	64 (94.1)
Break required for toxicity	12 (17.6)
Break duration [days, median (range)]	4 (2~26)
Concurrent chemotherapy	
Scheduled dose	54 (79.3)
Break required for toxicity	3 (4.4)
50%-90% of scheduled dose for toxicity	14 (20.7)
Omitted surgery	20 (29.4)
Refusal	7 (10.3)
Unresectable	6 (8.8)
Metastasis occurred before operation	3 (4.4)
Wait-and-see for CCR	2 (2.9)
Confirmation of lung cancer	1 (1.5)
Adverse event during anesthesia	1 (1.5)

*Abbreviations: CRT* Chemoradiotherapy, *RT* Radiotherapy, *CCR* Complete clinical response

### Oncological outcome

**Fig. 2:**
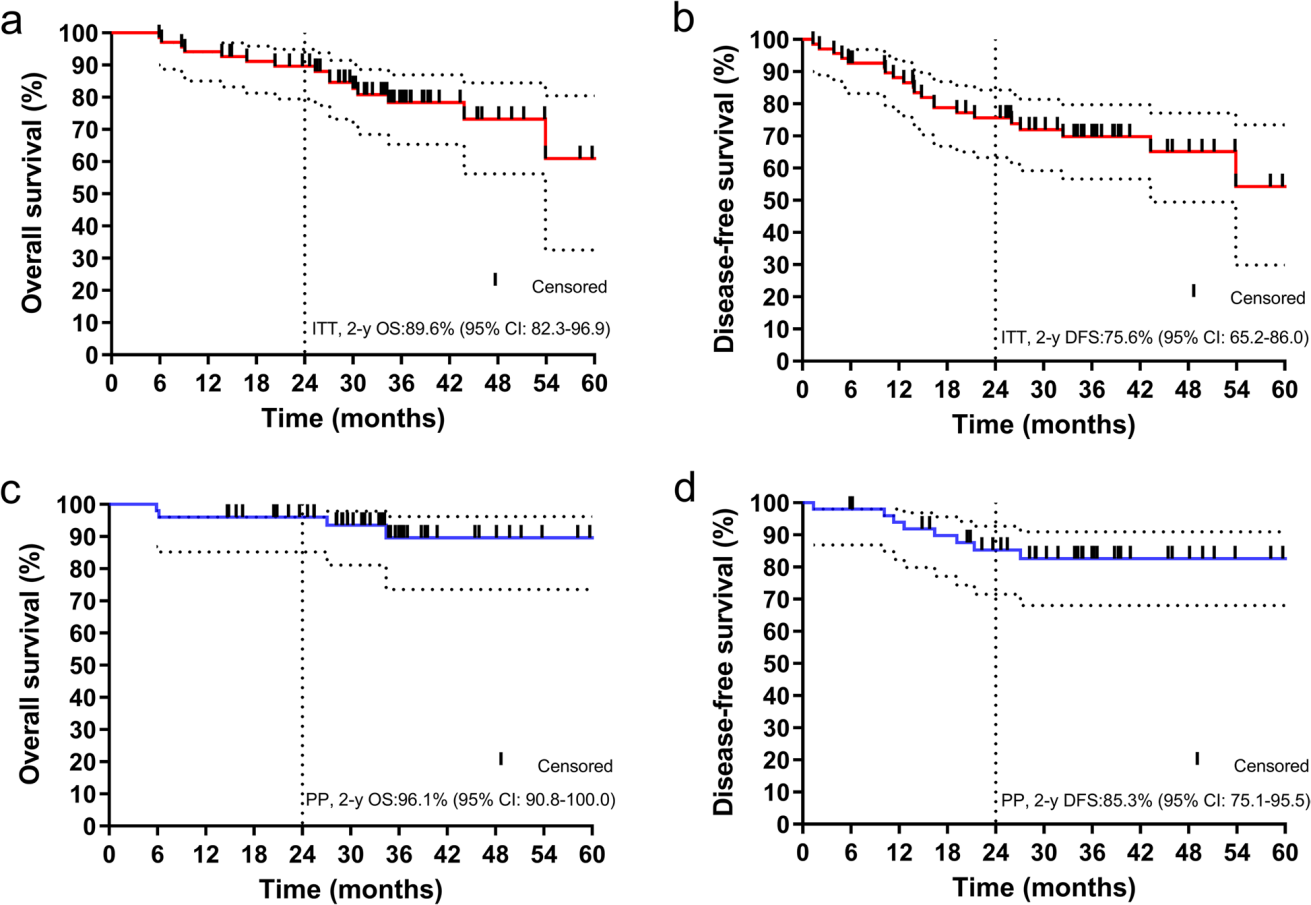
Survival curve by Kaplan-Meier method for ITT and PP populations. a, Overall survival for the ITT population; b, Disease free survival for the ITT population; c, Overall survival for the per-protocol population; d, Disease free survival for the per-protocol population

Overall, 48 patients underwent surgery, with a median interval between preCRT and surgery of 9.1 weeks (range: 5.6-104.1 weeks). The pathological response and evaluation are shown in Table S1. Among 47 positive mesorectal fascia (MRF+) patients, 36 (76.6%) received R0 resection. Three patient (4.4%) was lost to follow-up during a median of 36.0 months (range: 5.9-63.1 months); among the ITT population, eight local progression (2 recurrence included), fourteen metastases and fifteen deaths were observed; therefore, the primary end-point, the 2-year DFS was 75.6% (95% CI: 65.2-86.0), and the 2-year OS, CSS were 89.6% (95% CI: 82.3-96.9), and 92.4% (95% CI: 85.9-98.9), respectively. In the PP population (51), two local recurrence, nine metastases and four deaths were observed; therefore, the 2-year DFS, was 85.3% (95% CI: 75.1-95.5) (Fig. 2), and the 2-year OS, and CSS were 96.1% (95% CI: 90.8-100.0), and 98.0% (95% CI:94.1-100.0), respectively.

### Safety

Overall, the treatment demonstrated acceptable tolerability among patients, with 67 individuals (98.5%) experiencing some form of toxicity. For more details, please refer to Table 4. Among these, 24 patients (35.3%) experienced grade 3-4 side effects, while fourteen patients (20.6%) reported non-hematological side effects, with leukopenia, diarrhea, and fatigue being the most common. Additionally, five surgery-related complications were identified in five patients, resulting in a postoperative complication rate of 10.4%. According to the Clavien-Dindo classification system, there were one Grade I (intestinal obstruction), one Grade II (dysuria), one Grade IIIb (poor wound healing requiring skin grafting), one Grade IV (stroke with residual functional hemiparesis) and one Grade V (death within one week after surgery) complication. At 6 months of follow-up, the 30-day and 6-month mortality rates were both 1.5% (*n*=1).

**Table 4:** CRT toxicity

Toxicity	All grade (No., %) (*N*=68)	Grade 3-4 (No., %) (*N*=68)
Overall	67 (98.5%)	24 (35.3)
Hematologic		
Leukopenia	41 (60.3)	12 (17.7)
Anemia	7 (10.3)	-
Thrombocytopenia	2 (2.9)	-
Non-hematologic		
Anorexia	29 (42.7)	1 (1.5)
Fatigue	27 (31.8)	4 (5.9)
Diarrhea	21 (30.8)	7 (10.3)
Radiation dermatitis	18 (26.5)	1 (1.5)
Transaminase elevation	12 (17.6)	2 (2.9)
Pain	24 (35.3)	-
Proctitis	19 (27.9)	1(1.5)
Weight loss	9 (10.6)	-
Fever	4 (5.9)	1(1.5)
Vomiting	2 (2.9)	-
Urinary frequency	3 (4.4)	-
Hand-foot syndrome	-	1 (1.5)
Cardiac events	1 (1.5)	-

*Abbreviations: CRT* Chemoradiotherapy

### Exploratory endpoints

Important clinical factors, including sex, age, MRF status, social support scores and especially patient and family willingness, were analyzed for 56 surgery candidates evaluated by MDT after preCRT. The only significant factor that could predict surgery compliance was the patient’s own willingness to undergo treatment (relative risk 0.2, 95% CI 0.07-0.58, *P*=0.003). The likelihood of receiving surgery was 0.2 of the antecedent value when the score decreased by one point.

## Discussion

Despite current guidelines [10] and consensuses [3] recommending that “fit” older rectal cancer patients receive the same regimen as their younger counterparts, this concept still awaits verification in more prospective trials, especially the specific repeatable standard of frailty for adjusting the intervention. This study is the first exploration of CGA-driven stratified therapy based on the SIOG1 standard in older patients with LARC to evaluate the safety and efficacy of preCRT combined with the total mesorectal excision (TME) for a “fit” population. Through the ITT regimen, comparable oncological results were achieved between these older individuals and their younger counterparts from our STELLAR trial (about 75% 2-year DFS) during the same period of time [27], and the overall toxicity profile was generally tolerable. The primary endpoint of the study was met in the context of an effective and regular MDT; hence, the concept originating from guidelines and consensuses was validated. Moreover, a better understanding of the specific problem and challenges for older rectal cancer patients was provided in a quantitative geriatric assessment system.

In the general population with LARC, a personalized neoadjuvant strategy can be properly conducted according to tumor risk stratification [10]; unfortunately, the utilization of these approaches is severely restricted in patients of advanced age. Recent total neoadjuvant therapy (TNT) studies have demonstrated an improved DFS and more pCR [28, 29], but more toxicities and no improvement in OS were observed. This has complicated clinical decision making about neoadjuvant therapy in older patients with high-risk LARC. Short-course preoperative radiotherapy (SCPRT) followed by delayed surgery has greater potential for clinical application in a wider older population without limitation for geriatric status [8]. However, the final results of the PRODIGE 42/GERICO 12 study indicated that the non-inferiority in R0 resection rate was not achieved through SCPRT (25 Gy, 5 Gy/f) with delayed surgery comparing to the preCRT (50 Gy, 2 Gy/fraction + capecitabine) (R0 resection rate: 84.3% vs. 88.0%) in patients aged >75 years with WHO physical status (PS) ≤2 [8]. Furthermore, CGA and MRF status were, notably, not used in that study for inclusion criteria or stratification tool (at least, not reported), which may be the reason for the intragroup heterogeneity in the physiological state and the resectability of tumor, making it difficult to extract accurate information on the balance of benefit-risk. Moreover, compared with preCRT, SCPRT was inferior in reaching pCR (11.8% pCR was achieved in Stockholm III and approximately 15-20% in most preCRT studies) [30–33] and resectability conversion for MRF+ patients [10]. In summary, preCRT was utilized in the design of this study.

Given that 2-year DFS is a stronger predictor for OS than pCR [26], this surrogate was selected as the primary endpoint in the current study rather than pCR. Compared with the 75-80% 2-year DFS observed in several RCTs [28, 29, 34], the 2-year DFS (75.6%) in the older patients of this study was quite satisfactory. “Fit” older patients have a low burden of comorbidities and are in good physiological and psychological status; thus, a previous study in colorectal cancer showed that the 3-year noncancer mortality was <2%. Hence, the correlation between the 2-year DFS and OS in this study can be expected in longer follow-up, because the 3-year noncancer mortality was only 1.9%.

Even though the pCR rate in this study might appear to be low, it is still substantial considering that 69.1% of patients were MRF+, comparing with 12.3% of pCR rate was obtained in the control arm from contemporaneous STELLAR trial with 56.2% of patients were MRF+ [27]. Coupled with 76.6% of R0 resection rate for MRF+ patients, overall, the clinical application value of the current results for preCRT merits further research in “fit” older patients, especially for those patients with high-risk factors presented in RAPIDO [28]. From the perspective of greater surgery-related risk in advanced age patients, it may be prudent to explore some highly intensified treatments (TNT- or MRI-guided tumor boost) when the potential benefit of organ preservation outweighs their risks.

With respect to safety, compared with other studies, although the frequency of G3 or G4 acute toxicities induced by preCRT in this study was evidently higher than that in a younger population [19] and seemed to be slightly higher than that in older people [6], the majority of these toxicities were easy to handle, which is consistent with our previous interim analysis [22]. It is worth emphasising that most of the previous studies on older individuals were retrospective. Although the subgroup analysis for the older patients by Francois et al was from a randomized trial[6], the geriatric assessment tool was not employed as a quantitative standard. Different chemotherapy regimens and lack of CGA in earlier studies make it difficult to compare their findings with those of our study. In addition, the 79.3% completion rate for concurrent chemotherapy appeared to be greater than the scheduled dose delivery rate of 43.6% in a study from Francois [6] (half of the patients received a combination of capecitabine and oxaliplatin). It is worth mentioning that 32% of G3-5 toxicities in pre-operative phase were observed in chemoradiotherapy group of PRODIGE 42/GERICO study [8]. On the other hand, although all the patients in the preliminary results from PRODIGE 42/GERICO 12 [35] and half of the participants in the current study were aged 75 years and older, respectively, the 6-month mortality was much lower in our study (1.7% vs. 10%). The SIOG1 standard has high discriminability for 1-year mortality [36] and thus contributed more to the safety of this study. Consequently, considering the similar frequency of acute toxicities found in the recently conducted PRODIGE 42/GERICO study [8], which was specifically designed for older patients, it appears that the toxicity of the current regimen is tolerable and could serve as a reference for future studies. However, the drug selection in this study does not offer any additional advantages in terms of acute toxicity.

This study finally confirms the previous finding in our interim analysis [22], in which a relatively high proportion of older patients declined surgery. Rationally, it is not surprising that a large number of older patients do not aggressively advocate for surgery, especially in a multiple treatment setting [37]. However, it is still very important to study the factors related to surgery refusal, through a systematic review by Puts et al, it is known that factors affecting treatment decisions in older cancer patients varied considerably [38]. But more data regarding rectal cancer patients are urgently needed, because prediction of surgery omission may provide an opportunity for timely conduction boost by contact X-ray brachytherapy [39] or local excision [40]. In agreement with our previous report [22], the final result confirmed a role of the willingness evaluation of patients in predicting the compliance with surgery, which should be employed and validated in future investigations. Even more comprehensive communication with patients and training for physicians [41] may be needed in the shared decision-making era.

The main limitations of the current study include the single-arm design and relatively short follow-up time. In addition to the aforementioned shortcomings, due to the fact that the surgeons and patients were both more cautious for safety when confronted with advanced age and potential vulnerability, 80.1% of the patients included in our study had tumors with high-risk features. This may limit the extrapolation of the results to the older general population with LARC. On the other hand, patient-reported outcomes were lacking in the current study.

## Conclusions

Our results confirmed that preCRT is an effective treatment with tolerable toxicities for fit older people with locally advanced rectal cancer. Implementation of CGA before development of a treatment strategy should be considered in future research.

### Supplementary Information

Supplementary Material 1.

## Data Availability

Research data are stored in an institutional repository and will be shared upon request to the corresponding author.
