# Supplementary material for: Preoperative chemoradiotherapy in older patients with rectal cancer guided by comprehensive geriatric assessment within a multidisciplinary team—a multicenter phase II trial
Source: BMC Geriatr. 2024 May 21;24:442. doi: 10.1186/s12877-024-05046-6 (PMC11106876; doi:10.1186/s12877-024-05046-6)
Supplement: Supplementary file 1 — Supplementary Material 1. [file 12877_2024_5046_MOESM1_ESM.doc]

Table S1. Surgery and tumor pathological response results

| Item | No. (%) (N=48) |
| --- | --- |
| Surgery Type |  |
| Abdominoperineal | 23 (47.9) |
| Low anterior | 24 (50.0) |
| Hartmann | 1 (2.1) |
| Surgery margin |  |
| R0  R1 | 46 (95.8)  2 (4.2) |
| Response graded by Deworak |  |
| TRG 4 | 6 (12.5) |
| TRG 3 | 15 (31.3) |
| TRG 2 | 20 (41.7) |
| TRG 1 | 7 (14.6) |
| Pathologic tumor stage |  |
| ypT0 | 6 (12.5) |
| ypT2 | 12 (25.0) |
| ypT3 | 29 (60.4) |
| ypT4b | 1 (2.1) |
| Pathologic lymph node stage |  |
| ypN0 | 29 (60.4) |
| ypN1a | 7 (14.6) |
| ypN1b | 6 (12.5) |
| ypN1c | 2 (4.2) |
| ypN2a | 4 (8.3) |
| Pathologic complete response | 5 (10.4) |

Abbreviations: TRG, tumor regression grading.
